# Some Properties of the Mitochondria of Tumour Cells

**DOI:** 10.1038/bjc.1957.72

**Published:** 1957-12

**Authors:** V. Mutolo, F. Abrignani


### SOME PROPERTIES OF THE MITOCHONDRIA OF TUMOUR CELLS

V. MUTOLO AND F. ABRIGNANI

*From the Laboratory of Biology, Centro Tumori, Palermo, Italy*

Received for publication August 6, 1957

IN a previous paper (Abrignani and Mutolo, 1957) it has been shown that mitochondria prepared from tumour cells when incubated with versene lose a greater amount of compounds absorbing maximally at 260 m$\mu$ than mitochondria from normal liver cells do. This observation has led us to suggest a possible lability of the membrane of tumour cell mitochondria. A similar suggestion has been recently presented by Emmelot and Brombacher (1957).

In the experiments to be reported in this paper the swelling properties of the mitochondria from tumour cells have been compared to those of mitochondria of normal liver cells. The differential response of the two kinds of mitochondria to detergents and trypsin has also been investigated.

The rationale behind these experiments was that should the membrane of tumour mitochondria be altered in its structure or chemical composition, this would probably reflect in a different response with respect to mitochondria from normal cells, to certain experimental conditions, as e.g. the lowering of the osmolarity of the suspending medium.

The results we have obtained support the view that such an alteration does in fact exist in the tumour mitochondria.

### MATERIAL AND METHODS

### *Preparation of mitochondrial suspensions*

Normal albino rats and rats bearing subcutaneous 256 Walker tumour were used in these experiments. The liver of normal rats and the solid tumours free of necrotic tissues were collected immediately after killing the animals and homogenized at 0° C. in a glass homogenizer with a teflon plunger. The homogenization was carried out in nine volumes of 0·44 M sucrose in 0·1 M citrate buffer pH 6·3 containing $10^{-3}$ M versene (ethylendiaminetetracetic acid, tetrasodium salt) according to Witter, Watson and Cottone (1955). Nuclei and cell debris were sedimented at 700 $\times$ g. for 10 minutes in the cold. The supernatant was then centrifuged at 10,000 $\times$ g. for 20 minutes (in the MSE refrigerated centrifuge) and the sedimented mitochondrial pellet was suspended in five volumes of 0·44 M sucrose in 0·1 citrate buffer pH 6·3 and centrifuged again at 24,000 $\times$ g. for 20 minutes. The final sediment was suspended in 0·5 M sucrose in 0·02 M tris(hydroxymethyl) aminomethane buffer at pH 7·4 making use of a homogenizer. For the suspension of liver mitochondria five volumes of sucrose were used whereas half this volume was used for the suspension of tumour mitochondria. The mitochondria were used immediately after preparation.

*Experimental procedure*

In the experiments to investigate the swelling of the mitochondria, 0·1 ml. of the suspension were added to 3·0 ml. of sucrose solutions ranging from 0·5 to 0·1 M (in 0·02 M tris buffer pH 7·4) and finally to 3·0 ml. buffer alone; the suspensions were kept at the room temperature (19–20° C.) for the duration of the experiments. Optical densities were read at 520 m$\mu$ in a Beckman Spectrofotometer starting 30 seconds after mixing and then after 2, 5, 10, and 15 minutes.

In the experiments with versene, this was used at the final concentration of $5 \cdot 10^{-4}$ M.

For the study of the effect of detergents 0·1 ml. of the original mitochondria suspension were diluted in 3·1 ml. either of 0·3 M sucrose or of tris buffer containing 83 $\mu$g. of Duponol or 0·86 $\mu$moles of sodium deoxycholate.

In the experiments with trypsin, 150 $\mu$g. of crystalline (Armour) trypsin were added to 3·2 ml. of the mitochondria suspension in 0·3 M sucrose or in tris buffer.

No agglutination was ever observed in any one of the experiments.

### RESULTS

*Swelling experiments*

These experiments have shown that the swelling properties of tumour mitochondria in hypotonic solutions are considerably different from those of liver mitochondria.

Fig. 1 indicates that liver mitochondria begin to swell at 0·3 M sucrose. By further lowering the sucrose concentration a parallel increase of the rate of swelling of the mitochondria takes place, as indicated by the rapid decrease of the optical density of the suspension at 520 m$\mu$. The results are in good agreement with those of Tapley (1956).

Fig. 2 shows the changes in optical density of suspensions of tumour mitochondria at different concentrations of sucrose. It appears clearly that under these conditions the swelling of tumour mitochondria is considerably less than that of liver mitochondria.

These experiments indicate that tumour mitochondria maintain a low water content in spite of strongly hypotonic conditions of the medium.

*Effect of versene on the swelling of mitochondria*

Our experiments confirm that a $5 \cdot 10^{-4}$ M final concentration of versene effectively counteracts the swelling of liver mitochondria in hypotonic solutions.

On the contrary, as indicated in Fig. 3, the same concentration of versene appears to promote the swelling of tumour mitochondria.

*Effect of detergents*

As can be seen in Fig. 4, in the presence of both Duponol and sodium deoxycholate the decrease of the optical density of a mitochondria suspension is considerably greater in the case of liver than on tumour mitochondria.

*Effect of trypsin*

As reported by Dianzani (1953) a 0·04 per cent solution of crystalline trypsin is able to cause considerable damage to isolated liver mitochondria. In our

FIG. 1.—Swelling of liver mitochrondria. Change in optical density (at 520 mμ) of a suspension of liver mitochondria in (A) 0·5 M sucrose ; in (B) 0·4 M sucrose ; in (C) 0·3 M sucrose ; in (D) 0·2 M sucrose ; in (E) 0·1 M sucrose ; in (F) 0·02 M tris buffer pH 7·4. Sucrose in 0·02 M tris buffer pH 7·4. Total volume of mixture was 3·1 ml. $T$ = 19–20° C.

FIG. 2.—Swelling of tumour mitochondria. Change in optical density (at 520 mμ) of a suspension of tumour mitochondria in (A) 0·5 M sucrose ; in (B) 0·4 M sucrose ; in (C) 0·3 M sucrose ; in (D) 0·2 M sucrose ; in (E) 0·1 M sucrose ; in (F) 0·02 M tris buffer pH 7·4. Sucrose in 0·02 M tris buffer pH 7·4. Total volume of mixture was 3·1 ml. $T$ = 19–20° C.

Fig. 3.—Effect of versene on the swelling of tumour mitochondria. Solid lines show the swelling of mitochondria in (D) 0·2 M sucrose (in 0·02 M tris buffer pH 7·4); in (E) 0·1 M sucrose (in 0·02 M tris buffer pH 7·4); in (F) 0·02 M tris buffer pH 7·4; broken lines with addition of $5 \cdot 10^{-4}$ M versene. Total volume of mixture was 3·1 ml. $T = 19$–$20°$ C.

Fig. 4.—Effect of detergents. Control liver mitochondria (○—○) in 0·3 M sucrose (in 0·02 M tris buffer pH 7·4); the same with addition of 83 μg. of Duponol (●—●); and with 0·86 μmoles of sodium deoxicholate (●---●). Control tumour mitochrondria (△—△) in 0·3 M sucrose (in 0·02 M tris buffer pH 7·4); the same with addition of 83 μg. of Duponol (▲—▲); and with 0·86 μmoles of sodium deoxicholate (▲---▲). Detergents were added after 15 min. of preincubation. Final volume of mixture was 3·2 ml. $T = 19$–$20°$ C.

experiments we have used concentrations of trypsin somewhat lower than those used by Dianzani as it is conceivable that relatively small differences in sensitivity to trypsin would be detected more likely at low rather than at high concentrations of the enzyme.

As shown in Fig. 5, suspensions of liver mitochondria incubated with trypsin in 0·3 M sucrose or in tris buffer show a slight increase of the optical density during the first 30 minutes ; a moderate lowering of the optical density occurs only after 60 minutes of incubation.

On the contrary, a marked decrease of the optical density takes place in suspensions of tumour mitochondria under the same conditions (Fig. 6).

### DISCUSSION

Thus far the investigation of the properties of tumour mitochondria have revealed a number of differences with respect to mitochondria from normal cells in regard to their morphology (Howatson and Ham, 1955), protein and enzymatic composition (Hogeboom and Schneider, 1951 ; Schneider, Hogeboom, Shelton and Striebich, 1953).

The findings reported in the present paper suggest the existence of structural and chemical differences between the mitochondria of a tumour and those of the liver. The swelling properties of the mitochondria under passive experimental conditions, i.e. in which presumably no metabolic activity is occurring, indicate that tumour mitochondria have a much more limited permeability to water.

A structural-chemical difference between the proteins of the membrane of the two kinds of mitochondria is further indicated by the results of the experiments with detergents and trypsin.

The fact that the proteins of the tumour are more susceptible to trypsin may indeed be taken as an indication either that lipids play a less important role in the structure of such mitochondria or that their proteins are in a condition which one would indicate as denaturated. The former interpretation seems to be substantiated by the much weaker effect which anionic detergents, known to be very active on lipoprotein structures, exert on tumour mitochondria.

The present authors are well aware that in order for these results to gain general value and significance the mitochondria of several kinds of tumours and of different organs should be examined. Investigations along these lines are, in fact, in progress at this laboratory.

### SUMMARY

Differences have been found between mitochondria from normal rat liver and Walker tumour.

The tumour mitochondria suspended in hypotonic solution have shown much less swelling than those of normal liver.

Tumour mitochondria prove to be susceptible to the action of detergents and to the effect of trypsin.

These results suggest that changes occur in mitochondria membrane of tumour.

We wish to thank Prof. A. Monroy for valuable advice and helpful discussions.

FIG. 5.—Effect of trypsin on liver mitochondria. Mitochondria were suspended in 0·3 M sucrose (in 0·02 M tris buffer pH 7·4) without trypsin (□—□) and with 150 μg. of trypsin (■—■); in 0·02 M tris buffer pH 7·4 without trypsin (○—○) and with 150 μg. of trypsin (●—●). Total volume of mixture was 3·2 ml. $T$ = 19–20° C.

FIG. 6.—Effect of trypsin on tumour mitochondria. Mitochondria were suspended in 0·3 M sucrose (in 0·02 M tris buffer pH 7·4) without trypsin (▽—▽) and with 150 μg. of trypsin (▼—▼); in 0·02 M tris buffer pH 7·4 without trypsin (△—△) and with 150 μg. of trypsin (▲—▲). Total volume of mixture was 3·2 ml. $T$ = 19–20° C.

This investigation was supported in part by a grant from the Lega italiana per la lotta contro i tumori.

### REFERENCES

ABRIGNANI, F. AND MUTOLO, V.—(1957) *Naturwissenschaften*, **44**, 354.
DIANZANI, M. U.—(1953) *Experientia*, **9**, 343.
EMMELOT, P. AND BROMBACHER, P. J.—(1957) *Biochim. biophys. Acta*, **23**, 435.
HOGEBOOM, G. H. AND SCHNEIDER, W. C.—(1951) *Science*, **113**, 355.
HOWATSON, A. F. AND HAM, A. W.—(1955) *Cancer Res.*, **15**, 62.
SCHNEIDER, W. C., HOGEBOOM, G. H., SHELTON, E. AND STRIEBICH, M. J.—(1953) *Ibid.*, **13**, 285.
TAPLEY, D. F.—(1956) *J. biol. Chem.*, **222**, 325.
WITTER, R. F., WATSON, M. L. AND COTTONE, M. A.—(1955) *J. Biophys. Biochem. Cytol.*, **1**, 127.


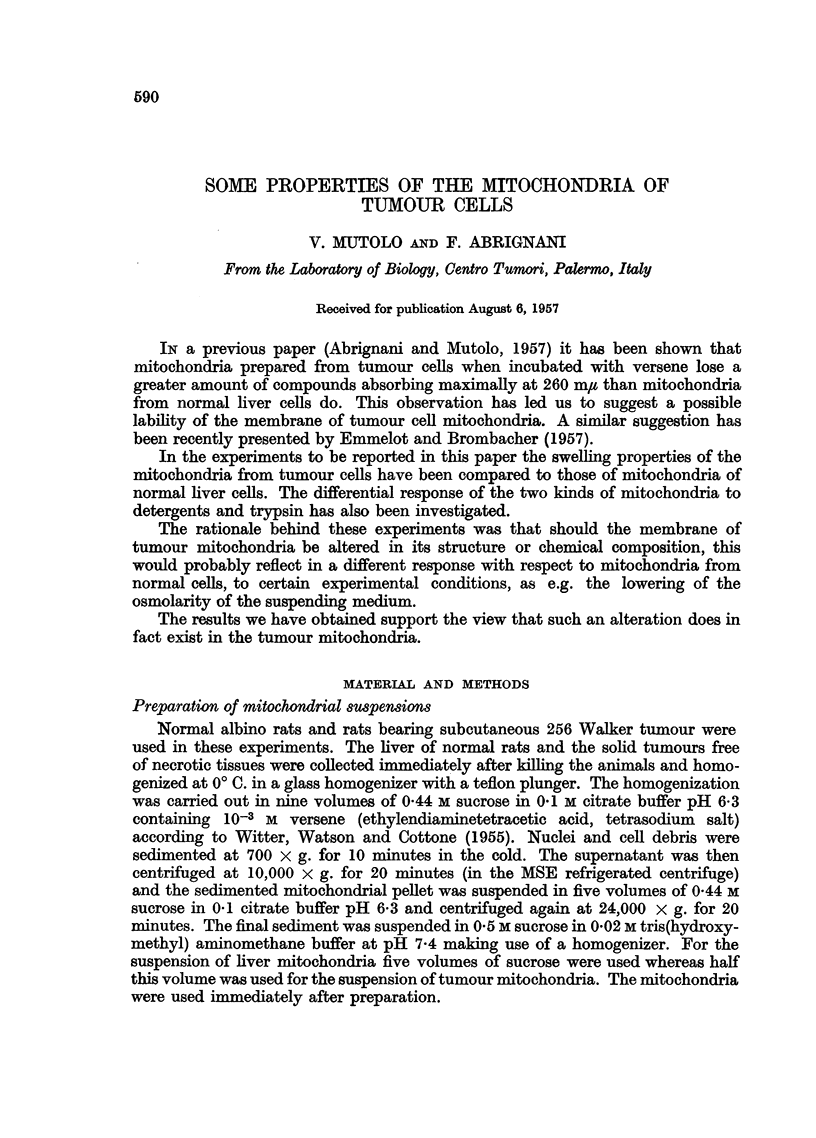

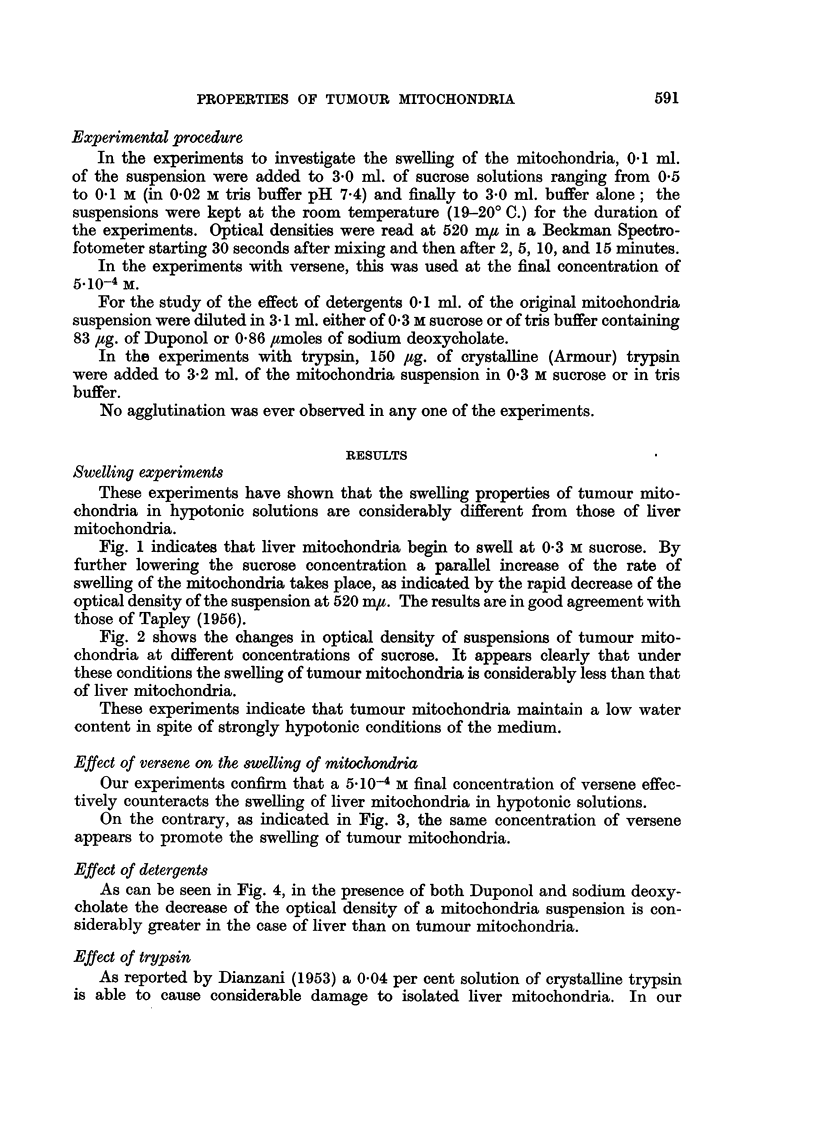

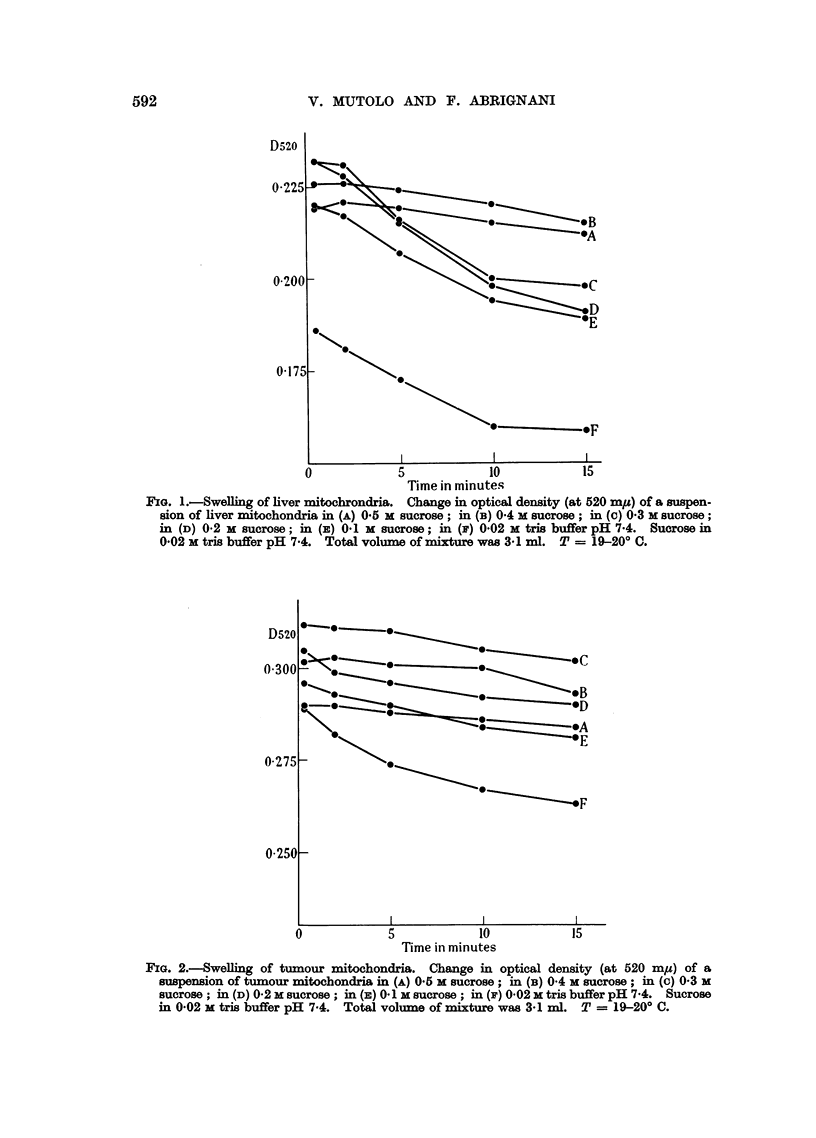

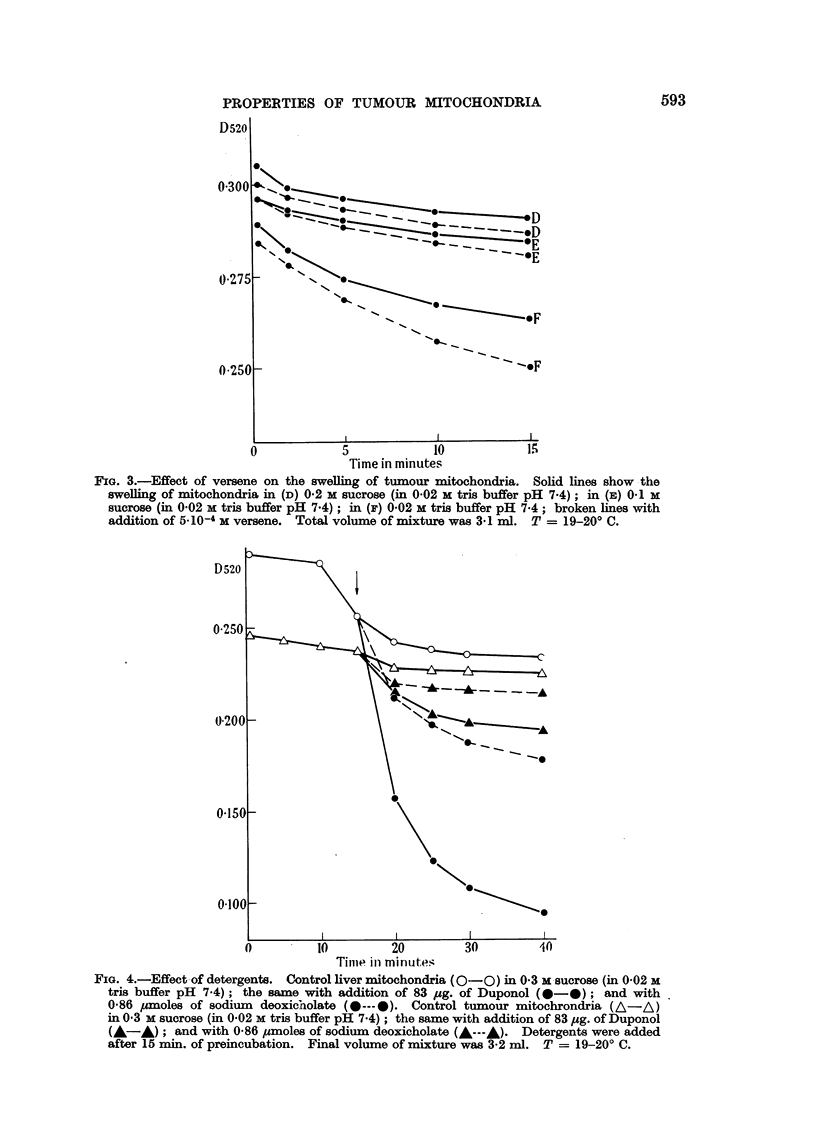

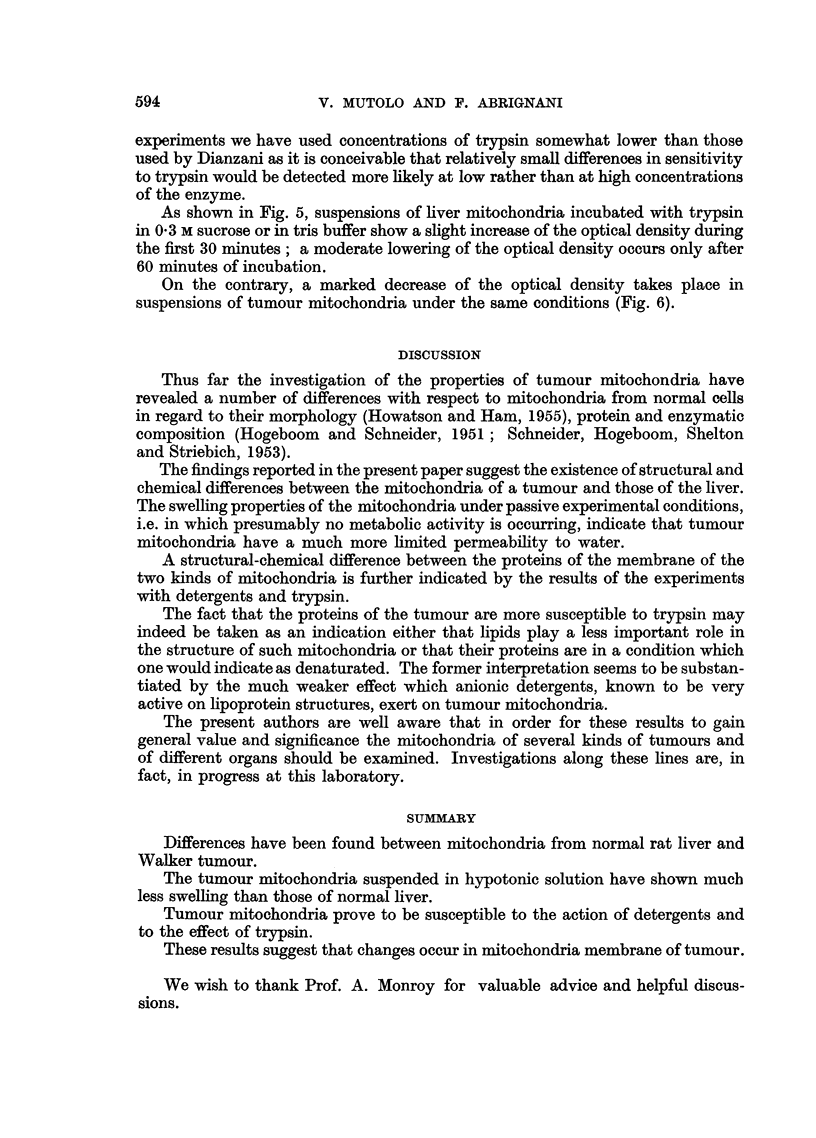

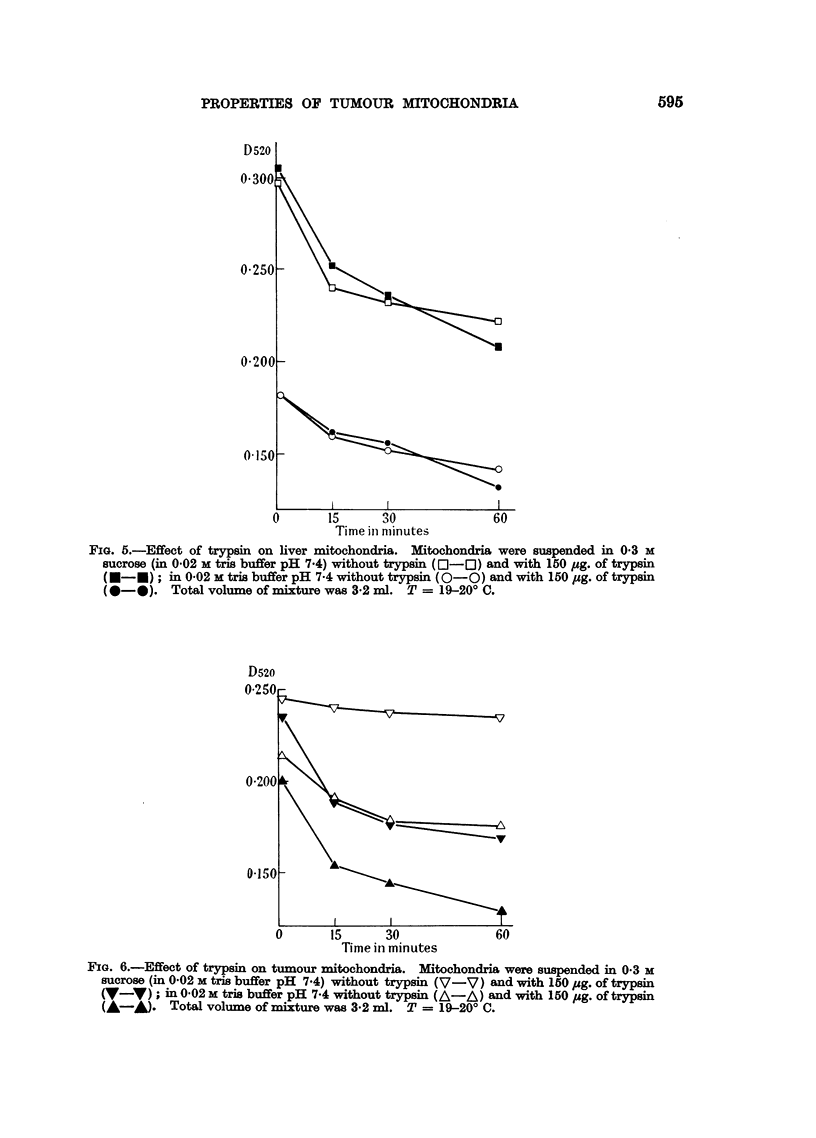

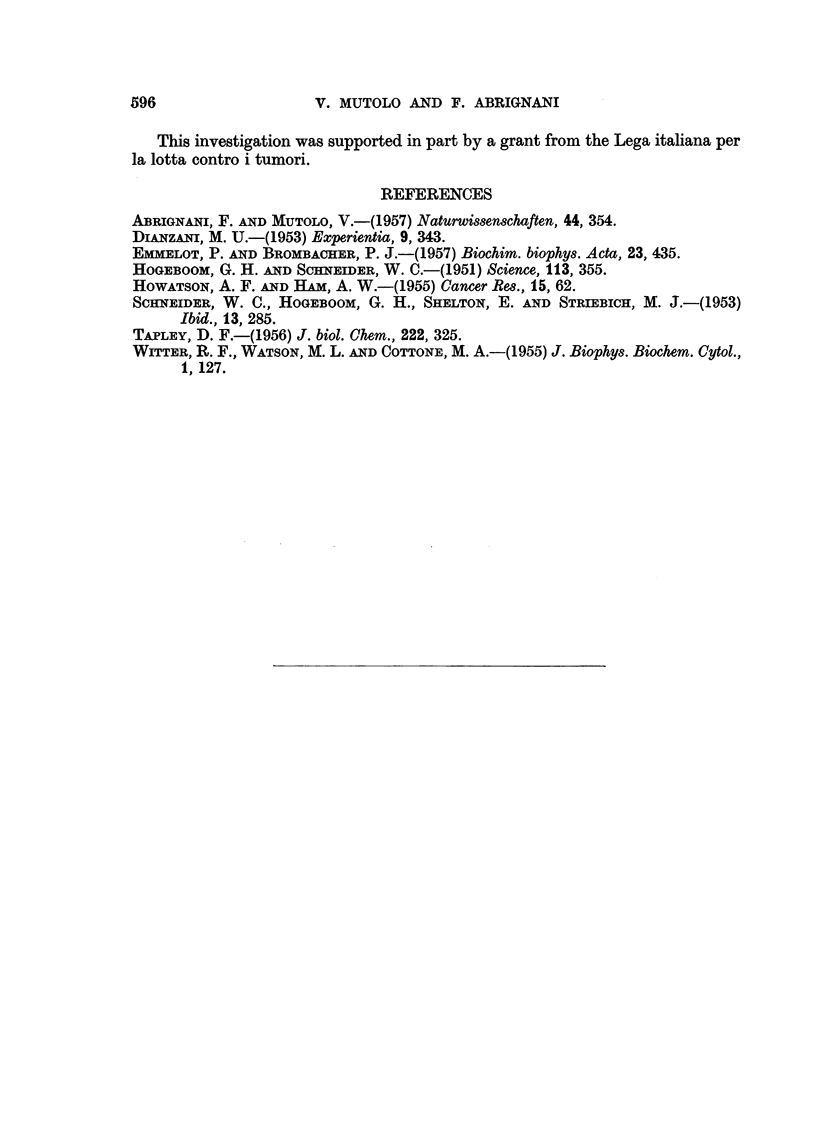


## References

[OCR_00290] EMMELOT P., BROMBACHER P. J. (1957). The effect of thyroxine on the oxidative phosphorylation of tumour mitochondria.. Biochim Biophys Acta.

[OCR_00291] HOGEBOOM G. H., SCHNEIDER W. C. (1951). Proteins of liver and hepatoma mitochondria.. Science.

[OCR_00294] SCHNEIDER W. C., HOGEBOOM G. H., SHELTON E., STRIEBICH M. J. (1953). Enzymatic and chemical studies on the livers and liver mitochondria of rats fed 2-methyl- or 3-methyl-4-dimethylaminoazobenzene.. Cancer Res.

[OCR_00298] TAPLEY D. F. (1956). The effect of thyroxine and other substances on the swelling of isolated rat liver mitochondria.. J Biol Chem.

[OCR_00300] WITTER R. F., WATSON M. L., COTTONE M. A. (1955). Morphology and ATP-ase of isolated mitochondria.. J Biophys Biochem Cytol.

